# Survey datasets on organisational climate and job satisfaction among academic staff in some selected private universities in Southwest Nigeria

**DOI:** 10.1016/j.dib.2018.06.001

**Published:** 2018-06-15

**Authors:** Anthonia Adeniji, Odunayo Salau, Kayode Awe, Olumuyiwa Oludayo

**Affiliations:** Covenant University, Nigeria

## Abstract

This study attempts to establish the relationships that exist between the different variables of organizational climate and job satisfaction among academic staff in some selected private Universities in South-West Nigeria, to ascertain related factors in organizational climate that can cause dissatisfaction among academics; and to determine if there is a significant difference in the way senior academics and junior academics perceive the existing organizational climate. A total of 384 copies of questionnaires were administered to selected five (5) private Universities in the South-West Zone of Nigeria but a total of 293 questionnaires were returned fully and appropriately filled. The study made use of appropriate statistics such as measurement model (Confirmatory Factor Analysis) and Multiple Regression to obtain results.

**Specifications Table**

**Table:** 

Subject Area	Industrial Relations and Human Resource Management
More specific subject area	Job Satisfaction.
Type of data	Tables and Text files
How data was acquired	Field survey
Data format	Raw
Experimental factors	Administration of questionnaire (384) to selected five (5) private Universities in the Southwest Zone of Nigeria to establish the relationships that exist between the different variables of organisational climate and job satisfaction among academic staff.
Data source location	Nigeria
Data accessibility	Every data is attached to this article.

**Value of the Data**

• The data can produce useful highlight on the factors that university lecturers view as enhancing job satisfaction within the organizational climate.
• The management of schools will find the data helpful in improving staff morale and bringing about job satisfaction of their employees.
• The data will be of great value in recommending policies and strategies for mitigating organizational correlates of job dissatisfaction.
• To help in gaining understanding that the climates of an organization and job satisfaction vary together.
• The questionnaire attached can be modified, adopted or adapted for further comparative researches in private and public universities and other industries aside from educational industry.

## 1 Data

Survey method was used mainly by questionnaire to collect the data from University lecturers in Southwest Nigeria. Respondents were requested to respond to questions with self-administered and structured questionnaire. The researcher utilized one structured questionnaire for both the senior academics and junior academics. This was presented personally to all respondents by the researcher in the sampled universities. This enhanced uniformity of response bearing in mind the degree of variations in perception of what the organizational climate may be referred to [1].

The study populations from which the sample was drawn consist of eighteen (18) private universities in the Southwest Nigeria. Out of these private universities, five (5) were taken as the study sample through judgmental sampling method and questionnaires were administered to the academic staff ranging from the Professors, Associate Professors, Senior lecturers, Lecturers 1, Lecturers 2, Assistant lecturers and Graduate Assistants. The total number of academic staff in the selected private universities is 754. The private universities chosen for this study are Covenant University, Bells University of Technology, Crawford University, Babcock University and Bowen University.

## 2 Experimental design, materials and methods

The evolving competition in the higher education environment in Nigeria brought about by increase in the number of new Universities has necessitated the need for good organisational climate that will enable these Universities retain their best employees. Reports by NUC (2008) revealed that though Universities are increasing, yet the number of qualified teachers is not increasing proportionately. Thus, surveys are necessary to establish the relationships that exists between the different variables of organisational climate and job satisfaction among academic staff of selected private Universities in Southwest Nigeria.

Out of 384 copies of questionnaire administered, only 293 copies of questionnaires were returned representing 76.30%. Majority of the questions used were adapted with some modifications from a job satisfaction questionnaire. Questionnaire for the study were sorted and those that were not properly filled were removed. To minimize errors, data from questionnaire were coded so as to pave way for editing of data before the use of SPSS-Statistical package for Social Sciences-software.

For the purpose of efficiency and thoroughness two field assistants were recruited and trained. The training focused on the pertinent objectives and importance of the study, how to administer/conduct the study instruments and how to secure respondents’ informed consent. The researchers ensured that respondents were well informed about the study and the objectives of this research and they were encouraged with the participation process. Respondents were offered the opportunity to stay anonymous and their responses were treated confidentially.

Hence, this study has extensive implications for the institutions, academic staff, government, educators and researchers in this regard. It can be concluded that the success of these universities depend on the ability to impact on the motivation and job satisfaction of academic staff with a wide range of benefits to promote retention and reduce job-hopping. To this end, the data presented in this article is imperative for more comprehensive analysis as presented in Tables 1, 2, 3 and 4 respectively.

**Table 1:** Confirmatory factor analysis.

Sn.	Variable	Standard Factor Loading	Cronbach α	NNFI	CFI	SRMR	RMSEA	$\propto^{2}\left(\partial f.p-\mathrm{value}\right)$
1.	**Management and Leadership**	> 0.6	> 0.7	> .90	> .90	< 1		
	Management and leadership style in my University does not support lecturing profession.	.72	.896	0.95	0.94	0.05	0.10	224.18
	Senior academics do not provide feedback on employees’ evaluation and performance.	.78	.897	0.92	0.95	0.07	0.12	79.46
	I will like my Head of Department to change his or her leadership style.	.75	.897	0.96	0.95	0.06	0.11	124.65
2.	**Participation in Decision-making**		.893	0.93	0.93	0.08	0.08	342.78
	Junior academics participate in decision making.	.72	.891	0.91	0.93	0.06	0.09	138.78
	I am allowed autonomy in discharging my duties.	.74	.889	0.92	0.94	0.05	0.08	115.43
	My abilities are taken into consideration when delegating.	.77	.889	0.93	0.96	0.06	0.09	115.36
3.	**Challenging Job**		.890	0.91	0.92	0.08	0.09	510.38
	I believe that the University sets high standard of performance.	.80	.892	0.90	0.93	0.09	0.11	382.31
	Delegated responsibilities allowed me to overcome limitation in my experience.	.92	.893	0.90	0.94	0.07	0.09	358.92
	I find delegated responsibilities interesting.	.86	.893	0.92	0.94	0.09	0.10	386.13
4.	**Boredom and Frustration**		.894	0.94	0.96	0.09	0.09	261.17
	Lecturers are given sufficient instruction on how to go about their work.	.83	.892	0.92	0.94	0.08	0.08	95.39
	Senior academics schedule work for all categories of lecturers.	.81	.897	0.92	0.94	0.09	0.09	324.96
5.	**Fringe Benefits**							
	I am satisfied with the benefits that I receive at the University.	.85	.891	0.91	0.92	0.07	0.11	173.10
	The benefits I receive are adequate to fulfill my basic needs.	.92	.891	0.92	0.93	0.08	0.12	213.24
	The benefits in my University are equal with the external labour market.	.80	.890	0.92	0.94	0.08	0.10	189.16
6.	**Personnel Policies**							
	I am informed about any new or revised policies.	.87	.889	0.96	0.98	0.08	0.10	111.86
	I believe my departmental policies facilitate the achievement of my goals.	.92	.888	0.95	0.98	0.07	0.10	110.50
	My University sponsor local and overseas training.	.83	.888	0.96	0.99	0.08	0.10	121.14
7.	**Working Condition**							
	My department provides sufficient material for our use.	.96	.889	0.90	0.94	0.09	0.10	138.85
	I am facilitated to overcome limitations in my experience.	.97	.889	0.93	0.96	0.04	0.11	129.13
	My senior colleagues create a challenging environment for me.	.90	.889	0.92	0.95	0.05	0.09	126.01
	The University provides the equipment and resources necessary for me to execute my responsibilities.	.92	.888	0.93	0.95	0.06	0.09	86.02
	
8.	**Suitable Career Ladder**							
	Senior academics share useful information with junior academics.	.97	.888	0.90	0.94	0.08	0.09	132.92
	Senior academics ensure high performance among the junior academics.	.99	.888	0.90	0.94	0.09	0.10	108.24
	Senior academics provide me with opportunities to overcome any limitations in knowledge.	.98	.889	0.90	0.92	0.08	0.10	111.25
	I believe that I have opportunity for career advancement.	.90	.890	0.91	0.93	0.07	0.09	237.72
								
	**Appropriate Admin Style**							
	We spend too much time in meetings.	.85	.893	0.90	0.93	0.05	0.10	173.21
	Time spent in meetings keep me from doing my best on the job.	.91	.897	0.91	0.94	0.06	0.10	80.74
	If I have my way, I will avoid going for the meetings.	.84	.897	0.93	0.96	0.05	0.10	79.67
	**Support from Supervisors**							
	Senior academics help to solve personal problems of their junior colleagues.	.86	.889	0.95	0.98	0.07	0.10	141.41
	Senior academics sometimes do personal favour for junior academics.	.94	.888	0.93	0.96	0.06	0.10	136.63
	Senior academics encourage their subordinates to take initiatives in solving problems.	.97	.888	0.94	0.97	0.05	0.10	129.23
	Senior academics are willing to listen to job related problems.	.80	.889	0.92	0.99	0.04	0.10	130.36
	**Work load**							
	Courses allocated to me are sometimes outside my area/field of specialization.	.91	.896	0.92	0.94	0.07	0.09	86.69
	My workload is often increased because my colleagues are not doing their jobs properly.	.90	.895	0.92	0.96	0.07	0.08	85.24
	My level of education and experience is used in allocating courses.	.81	.893	0.91	0.92	0.07	0.09	244.68
	**Feedback Performance**							
	Senior academics explain reasons for his or her criticism.	.87	.888	0.94	0.96	0.09	0.10	123.75
	I am promoted based on my performance.	.71	.892	0.95	0.99	0.08	0.11	237.93
	My performance appraisal are fair.	.96	.889	0.90	0.92	0.07	0.10	95.97
	**Clear Lines of Communication**							
	I am made aware of the rules and regulations I have to follow.	.97	.889	0.91	0.92	0.08	0.11	170.09
	It is easy for me to talk with my superior.	.91	.888	0.94	0.96	0.08	0.10	135.26
	I know exactly what is expected of me.	.96	.890	0.92	0.98	0.09	0.10	217.72

**Table 2:** Model summary of organisational climate and satisfaction.

**Model**	**R**	**R Square**	**Adjusted R Square**	**Std. Error of the Estimate**
1	.908(a)	.825	.823	.20318

a Predictors: (Constant), PROMOOPP, SALARYPACK, COMMUNICATN

b Dependent Variable: JOBSATIS

**Table 3:** ANOVA of organisational climate and satisfaction.

Model		Sum of Squares	Df	Mean Square	F	Sig.
1	Regression	56.167	3	18.722	453.524	.000(a)
	Residual	11.931	289	.041		
	Total	68.098	292			

a Predictors: (Constant), PROMOOPP, SALARYPACK, COMMUNICATN

b Dependent Variable: JOBSATIS

**Table 4:** Summary of estimated coefficients of organisational climate indicators.

Model		Unstandardized Coefficients		Standardized coefficients	T	Sig.
		B	Std. Error	Beta	B	Std. Error
1	(Constant)	.994	.064		15.621	.000
	COMMUNICATN	.253	.019	.411	13.122	.000
	SALARYPACK	.172	.017	.274	10.401	.000
	PROMOOPP	.266	.019	.453	14.015	.000

a Dependent Variable: JOBSATIS
