# Supplementary material for: Survey datasets on organisational climate and job satisfaction among academic staff in some selected private universities in Southwest Nigeria
Source: Data Brief. 2018 Jun 15;19:1688–93. doi: 10.1016/j.dib.2018.06.001 (PMC6140823; doi:10.1016/j.dib.2018.06.001)
Supplement: Supplementary file 1 — Supplementary material [file mmc1.docx]

**DECLARATION OF INTEREST FORM**

**SURVEY DATASETS ON ORGANISATIONAL CLIMATE AND JOB SATISFACTION AMONG ACADEMIC STAFF IN SOME SELECTED PRIVATE UNIVERSITIES IN SOUTHWEST NIGERIA**

Anthonia **ADENIJI**; Covenant University

anthonia.adeniji[@covenantuniversity.edu.ng](mailto:olumuyiwa.oludayo@covenantuniversity.edu.ng)

Odunayo **SALAU**; Covenant University

odunayo.salau@covenantuniversity.edu.ng

Kayode **AWE**; Covenant University

kayode.awe[@covenantuniversity.edu.ng](mailto:olumuyiwa.oludayo@covenantuniversity.edu.ng)

Olumuyiwa **OLUDAYO**; Covenant University

[olumuyiwa.oludayo@covenantuniversity.edu.ng](mailto:olumuyiwa.oludayo@covenantuniversity.edu.ng)

We, the Authors of paper entitled above certify that we have seen and approved the final version of the manuscript being submitted. This is an original work and has not received prior publication and is not under consideration for publication elsewhere. It is also important to state that there is no financial/personal interest or belief that could affect our objectivity and to prevent ambiguity, we humbly want to state explicitly that there is no conflicts of interest as regards the review and publication of this paper.

Thank you.

ADENIJI Anthonia Adenike

*Signed*
